# Supplementary material for: Learning curve and surgical outcome of robotic assisted colorectal surgery with ERAS program
Source: Sci Rep. 2022 Nov 29;12:20566. doi: 10.1038/s41598-022-24665-w (PMC9709162; doi:10.1038/s41598-022-24665-w)
Supplement: Supplementary file 1 — Supplementary Information 1. [file 41598_2022_24665_MOESM1_ESM.zip › LSC Raw data-Ñ[▒K/138/O_ñw╝╨░O▒Kñσ.pdf]

正式摘要(電子簽章：000000225667999)

W92 / W92

臺中榮民總醫院

Operation Note

[redacted]

- 手術日期：2021/09/23
- 手術醫師：VS 蔣鋒帆，VS 邱騰逸，R 黃瑋，R 曾禹康
- Anesthesia : GE
- Position : Lithotomy
- Aseptic agents : Soap , Betadine Alcohol , Betadine Aqua

Diagnosis

- Pre-operative diagnosis : Malignant tumor of rectum
- Post-operative diagnosis : Ditto.

Surgical Indication

- to remove malignant neplasm for pathological diagnosis

Operation

- Low anterior resection with colo-anal anastomosis Ileostomy Laparoscopy

Finding

- 1. An proturded tumor, about 2cm x 3 cm in size, located at middle rectum, didn't penetrate through the serosa. No palpable lymph nodes at mesocolon, along intermediate vessel to IMA root.
  2. No visible liver metastasis or peritoneal seeding

Procedure

- 1. Under general anesthesia, the patient was placed in lithotomy position. The abdomen and perineum was prepped and draped in standard sterile fashion.
  2. A small incision was made near umbilicus and 12mm ballon troca was inserted. Pneumoperitoneum was established with CO2 inflation. Laparoscopy was inserted and then another 5mm and 12mm trochars were inserted over RUQ and RLQ respectively under laparoscopic guide. Another two 5mm trochars were inserted at LLQ and LUQ under laparoscopic guide.
  3. Mesosigmoid colon was hold by grasper and dissection was made to separate mesocolon from retroperitoneum and further dissection was done toward mesorectum.
  4. The IMA was identified, dissected, skeletonized by Harmonic scalpel and transected by triple clips and Harmonic energy device. The IMV was done in the same way.
  5. The mobilization of S-colon was done with dissection via embryonic plane between Gerota fascia and meso-sigmoid colon. The lateral attachement of sigmoid colon was also divided.
  6. After fully mobilization of S-colon, the rectum was also dissected posteriorly in upper rectum level, Denovillier fascia anteriorly. The bilateral lateral ligaments were divided
  7. Transection of rectum at peritoneal reflection was done by sixe 60 mm endocutters.
  8. A small transverse incision was made at LLQ. The specimen was pulled out. The 29 mm envil was inserted in proximal colon and put back into abdominal cavity.
  9. Distal irrigation via anus was done.
  10. The end to end colorectal anastomosis was done by double stappling technique.
  11. One 19Fr hemovac drainage was placed at pelvis.
  12. The transverse wound, paraumbilicus wound, RLQ wound were closed with No.1 Vicryl suture. All wounds were irrigated and closed with 3-0 nylon.
  13. Under stable condition, the patient was sent to POR.
- Estimated blood loss: 250 c.c
- 器械數量: 正確

- 紗布數量: 正確

主任 2062F蔣鋒帆 / 醫師 2338B黃瑋

正式摘要(電子簽章：000000225667999)

W92 / W92

doctorRank:B
